# Improving Microbiological Monitoring of Hospital Surfaces: Loop-Mediated Isothermal Amplification (LAMP) as a New Approach for Rapid Nosocomial Pathogens Detection

**DOI:** 10.3390/ijerph23020174

**Published:** 2026-01-30

**Authors:** Federica Marino, Caterina Bonincontro, Laura Caligaris, Derelitto Carlo, Luna Girolamini, Sandra Cristino

**Affiliations:** Department of Biological, Geological, and Environmental Sciences, University of Bologna, 40126 Bologna, Italy; federica.marino20@unibo.it (F.M.); caterina.bonincontr2@unibo.it (C.B.); laura.caligaris2@unibo.it (L.C.); carlo.derelitto@unibo.it (D.C.);

**Keywords:** hospital surfaces, LAMP, nosocomial pathogens, healthcare-associated infections, microbiological monitoring, rapid detection

## Abstract

**Highlights:**

**Public health relevance—How does this work relate to a public health issue?**

- Hospital surfaces represent a recognized source of nosocomial pathogens involved in healthcare-associated infections (HAIs).
- Gold-standard methods are time-consuming and may limit the effectiveness of infection prevention strategies.

**Public health significance—Why is this work of significance to public health?**

- This study shows that LAMP allows rapid and sensitive detection of major nosocomial pathogens on hospital surfaces.
- Short incubation times enable faster environmental surveillance compared with conventional culture-based methods.

**Public health implications—What are the key implications or messages for practitioners, policy makers and/or re-searchers in public health?**

- Rapid molecular screening of hospital surfaces can support timely infection control decisions and targeted cleaning interventions.
- Targeted cleaning interventions optimize resource allocation and reduce unnecessary sanitation costs.

**Abstract:**

Hospital environments are recognized as significant reservoirs of nosocomial pathogens, contributing to the onset of healthcare-associated infections (HAIs). Timely microbiological monitoring is essential to mitigate infection risks. However, gold-standard methods based on culture and biochemical techniques are time-consuming and may underestimate microbial contamination, potentially delaying interventions. This study proposes a novel approach for surface monitoring using loop-mediated isothermal amplification (LAMP) for the rapid detection of key nosocomial pathogens, such as *Pseudomonas aeruginosa*, *Staphylococcus aureus*, and *Enterococcus* spp. A total of 145 surface samples were collected from six Italian hospitals and analyzed by both standard culture and LAMP methods, following two different incubation times (6 and 9 h) using pre-enrichment medium. Comparison with the reference method revealed that the LAMP assay achieved a sensitivity of 1.00 for all target pathogens at both 6 and 9 h of incubation. Specificity values were slightly higher at 6 h compared to 9 h: 0.93 vs 0.90 for *P. aeruginosa*, 0.91 vs 0.89 for *Enterococcus* spp., while remaining 0.92 for *S. aureus*, at both incubation times. These results suggest that a 6-h incubation period offers an optimal balance between speed and diagnostic accuracy, making LAMP a promising tool for rapid microbiological surveillance in healthcare settings.

## 1. Introduction

Hospital environments can be considered important reservoirs of nosocomial pathogens capable of causing healthcare-associated infections (HAIs), which represent one of the major public health challenges worldwide due to their impact on morbidity, mortality, and healthcare costs [1].

According to the latest surveillance report from the European Centre for Disease Prevention and Control (ECDC), the estimated prevalence of HAIs in European hospitals is 6.5%, meaning that approximately 3.8 million patients are affected across Europe [2]. The clinical impact is significant, with HAIs responsible for over 90,000 deaths annually [3]. In addition, HAIs contribute to prolonged hospital stays, increased antimicrobial consumption, and greater resource utilization [4].

Growing evidence in the scientific literature highlights the role of environmental surfaces in the transmission of nosocomial pathogens, particularly in high-risk areas such as intensive care units and surgical wards [5]. In fact, several studies have already demonstrated that pathogens such as *Pseudomonas aeruginosa* (*P. aeruginosa*), *Staphylococcus aureus* (*S. aureus*), and *Enterococcus* spp. can be frequently isolated from hospital surfaces, mainly due to their ability to persist for extended periods under various environmental conditions [6,7,8]. Transmission can occur through direct contact with contaminated surfaces or indirectly via healthcare workers, patients, or visitors who come into contact with these surfaces, facilitating the spread of pathogens to other individuals or areas [9].

For this reason, routine microbiological monitoring of hospital surfaces is crucial for preventing and controlling infections in these highly sensitive environments, especially due to the presence of immunocompromised individuals who are more vulnerable to pathogenic bacteria [10]. Infection prevention and control measures, including environmental hygiene, hand hygiene, and surveillance, have been recognized by the World Health Organization (WHO) as a key strategy to improve patient safety and reduce the burden of HAIs [11]. However, the current gold-standard methods, based on culture and biochemical identification, are labour-intensive and time-consuming, often requiring several days for results [12,13]. Moreover, another limitation lies in the failure to detect viable but non-culturable (VBNC) organisms. These bacteria, although metabolically active and potentially infectious, fail to grow on standard laboratory media, leading to a systematic underestimation of environmental contamination levels [14]. Consequently, culture-based monitoring may provide a false sense of security, hindering the accurate assessment of the true microbial burden and delaying timely interventions [15].

In response to these limitations, in recent years, molecular techniques have increasingly been adopted as rapid and sensitive alternatives [16,17]. Among them, loop-mediated isothermal amplification (LAMP) has emerged as a particularly promising tool for rapid diagnostic [18]. Unlike conventional molecular methods, LAMP does not require complex instrumentation, as it operates under constant temperature conditions (typically between 60 and 65 °C) [19,20]. This feature not only simplifies the workflow, enabling faster turnaround times (often within 30 to 60 min), but also makes the method particularly suitable for field use, reducing both response time and the need to transport samples to centralized laboratories [21,22]. The amplification is driven by a strand-displacing DNA polymerase, which synthesizes DNA without the need for thermal cycling [23]. Its high specificity is ensured by the use of a set of four to six primers, which recognize six to eight distinct regions of the target DNA [24]. The reaction generates large amounts of DNA, detectable through turbidity or fluorescence, allowing for easy and rapid visualization of results [25].

Due to its characteristics, the use of LAMP has been extensively documented in several fields, including clinical diagnostics, veterinary medicine, and food safety control [26,27,28]. Its operational flexibility and adaptability to different matrices suggest that LAMP could be effectively tailored to hospital environmental monitoring. 

Building on previous findings obtained under laboratory-controlled conditions [29], the present study aimed to evaluate the applicability of LAMP for the rapid detection of *P. aeruginosa*, *S. aureus*, and *Enterococcus* spp. on hospital surfaces on site. Sensitivity, specificity, and practical applicability were assessed to determine the potential of LAMP as a rapid tool for improving microbiological surveillance and enabling timely infection control interventions in healthcare environments.

## 2. Materials and Methods

### 2.1. Sample Collection

A total of 145 surface samples were collected from six hospitals, targeting various high-touch areas with differing levels of infection risk. Sampling was performed using SRK^®^ FLOQSwabs^®^ (Copan, Brescia, Italy), according to the UNI EN 17141:2021 [30] standard for environmental surface monitoring.

Specifically, the samples were collected as follows:

20 samples from very high-risk areas (VHR), such as operating rooms and pre-surgical zones;

45 samples from high-risk areas (HR), including intensive care units and emergency departments;

65 samples from medium-risk areas (MR), such as patient rooms and general wards;

15 samples from low-risk areas (LR), including outpatient clinics and waiting rooms.

The sample size was determined based on a risk-based rationale. Low-risk (LR) areas were underrepresented, as they are typically designated for short patient stays and do not involve invasive procedures. These areas generally require only minimal disinfection, making them less relevant for evaluating the risk of infection transmission. Similarly, VHR areas were also sampled less frequently, as these environments undergo intensive disinfection protocols, reducing the likelihood of detecting positive samples. Moreover, collecting only negative samples would not allow for a meaningful evaluation of the diagnostic performance of the tested kits. Conversely, a greater number of samples were collected from HR and medium-risk MR areas, which were considered optimal environments for testing the kits due to their intermediate balance between infection risk and cleaning procedures.

For flat surfaces, a standardized area of 10 × 10 cm (100 cm^2^) was sampled using sterile masks to delimit the sampling zone precisely. For irregular surfaces, the sampled area was approximated using a disinfected measuring tape. To ensure complete coverage, each swab, once moistened, was passed over the surface in three different directions (vertical, horizontal, and diagonal), applying consistent pressure throughout the sampling process. The swab-based sampling method was selected for its compatibility with the experimental workflow involving LAMP analysis, particularly due to its efficiency in recovering microorganisms from surfaces and its suitability for downstream molecular applications.

Each sample was analyzed using both the gold-standard culture-based method, representative of the standard workflow prescribed by current regulatory guidelines [31,32], and the experimental LAMP approach, allowing for a direct comparison of sensitivity and specificity between the two techniques.

### 2.2. Culture-Based Analysis

Each swab was processed by plating 250 µL of the sample onto Tryptic Soy Agar (TSA) plates (Thermo Fisher Scientific, Diagnostic, Ltd., Basingstoke, UK) and incubated at 35 °C ± 2 °C for 72 h. At the end of the incubation period, all visible colonies were counted and grouped according to their morphological characteristics. For each distinct morphology, at least five representative colonies were selected for identification using the Matrix-Assisted Laser Desorption/Ionization Time of Flight (MALDI) Biotyper^®^ sirius System (Bruker Daltonics, Bremen, Germany).

The results were recorded and expressed as colony-forming units (CFU) per 250 µL of sample (CFU/250 µL), providing a quantitative measure of microbial contamination for each surface. However, since LAMP provides qualitative results, culture outcomes were also interpreted qualitatively; therefore, colony counts and species-level identifications were not reported, as they were not relevant to the performance evaluation.

### 2.3. LAMP Analysis

The remaining sample (2.25 mL) from each swab was incubated in a pre-enrichment medium, as recommended by the LAMP kit protocol, to enhance the recovery and amplification of target microorganisms. In line with the methodology previously explored by Marino et al. [29], an aliquot of 1.4 mL was taken from each sample after 6 and 9 h of incubation. This approach was adopted to evaluate which incubation time provided the best analytical performance in terms of sensitivity and specificity, and to determine the shortest possible time to obtain highly reliable results compared to the gold-standard method.

Following incubation, DNA extraction was performed according to the procedure suggested by the LAMP kit manufacturer (Enbiotech, Palermo, Italy). Each sample was vortexed for 10 s and centrifuged at 9500 *g* for 5 min. The supernatant was carefully removed, either by inversion or pipetting, without disturbing the pellet, inside a laminar flow biological safety cabinet. A total of 200 µL of extraction buffer, provided by the kit, was then added to the pellet, and the tube was vortexed for 30 s. The sample was incubated at 95 °C for 10 min, vortexed again for 10 s, and centrifuged at 13,000 *g* for 1 min to complete the extraction process.

The extracted DNA was then used to prepare three different LAMP reactions, using three pathogen-specific LAMP kits, targeting *P. aeruginosa*, *S. aureus*, and *Enterococcus* spp., respectively. All kits were manufactured and distributed by Enbiotech, Palermo, Italy. Each kit included all necessary components: Primer Mix tubes, LAMP Mix, and positive and negative controls.

For each sample, a corresponding Primer Mix tube was prepared and labeled with the sample code. The reaction mix was assembled as follows:

For *P. aeruginosa*: 14 µL of LAMP Mix and 6 µL of DNA;

For *S. aureus*: 14 µL of LAMP Mix and 6 µL of DNA;

For *Enterococcus* spp.: 22 µL of LAMP Mix and 3 µL of DNA.

DNA was pipetted carefully to facilitate the resuspension of lyophilized primers located at the bottom of each tube. Each run included positive and negative controls to ensure the reliability and reproducibility of the results. The *P. aeruginosa* and *S. aureus* LAMP kits include an internal amplification control to monitor reaction performance and detect potential polymerase inhibition. For *Enterococcus* spp., no separate internal control is included; however, the pre-enrichment step dilutes potential inhibitory residues from disinfected surfaces, minimizing the risk of false-negative results.

The data regarding the reagents provided in the kit, including the amplification primer sequences, are covered by the manufacturer’s intellectual property.

The amplification was performed at the constant temperature of 65 °C using the ICGENE Plus device (Enbiotech, Palermo, Italy), a portable real-time isothermal amplification system. The instrument was operated via a dedicated tablet equipped with the ICGENE application (ICGENE app version 3.9.10), which enabled run configuration and execution. After loading the samples into the designated slots and initiating the amplification process, the system automatically interpreted the results, providing a clear positive (‘+’) or negative (‘−’) outcome for each target pathogen.

### 2.4. Statistical Analysis

For each method (gold-standard culture, LAMP at 6 h, and LAMP at 9 h), the percentage of positive samples was determined for each of the three target microorganisms: *P. aeruginosa*, *S. aureus*, and *Enterococcus* spp.

To evaluate the diagnostic performance of the LAMP method, several performance metrics were calculated for each incubation time (6 and 9 h), including sensitivity (Se), specificity (Sp) with their respective 95% confidence intervals (95% CI), accuracy (Acc), positive predictive value (PPV), negative predictive value (NPV), F1-score (F1), and balanced accuracy (Bal Acc) [33]. These metrics were derived from contingency tables by comparing the results obtained with LAMP to those from the reference culture-based method, which was considered the gold standard.

In addition, the level of agreement between the LAMP results (at both incubation times) and the gold-standard method was assessed using Cohen’s kappa coefficient (κ), which provides a measure of concordance beyond chance [34].

## 3. Results

The analysis revealed that, among a total of 145 hospital surface samples, the culture-based gold-standard method identified *P. aeruginosa* in 6 samples (4%), *S. aureus* in 10 samples (7%), and *Enterococcus* spp. in 18 samples (12%).

In contrast, the LAMP method showed higher detection rates. After 6 h of incubation, *P. aeruginosa* was detected in 16 samples (11%), *S. aureus* in 21 samples (14%), and *Enterococcus* spp. in 30 samples (21%). After 9 h, the number of positive samples increased slightly: 20 for *P. aeruginosa* (14%), 21 for *S. aureus* (14%), and 32 for *Enterococcus* spp. (22%) (Table 1).

**Figure 1:**
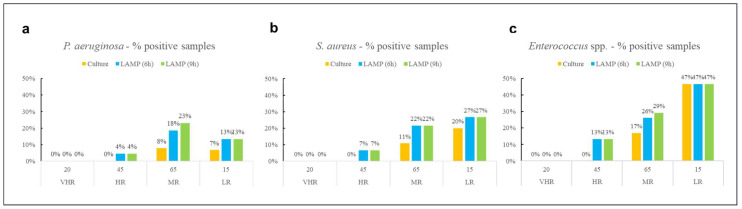
Percentage of positive samples for (**a**) *P. aeruginosa*, (**b**) *S. aureus*, and (**c**) *Enterococcus* spp. across different hospital risk areas and methods.

The percentage of positive samples for each microorganism was also analyzed according to the hospital risk areas (VHR, HR, MR, LR) and detection method (Figure 1). This stratification allowed for a more contextual interpretation of contamination levels.

No contamination was detected in VHR areas for any of the target pathogens, regardless of the detection method used. For *S. aureus* and *Enterococcus* spp., the highest proportion of positive samples across all methods used was observed in LR environments, while *P. aeruginosa* was most frequently detected in MR areas. The culture-based method identified all three pathogens exclusively in MR and LR areas, with no detections in other areas.

To evaluate the performance of the innovative LAMP method, for each kit studied, performance metrics were calculated after both 6 and 9 h of incubation with the pre-enrichment medium, using the gold standard culture-based method as the reference (Table 2).

Data showed that Se and NPV were equal to 1.00 for all detection kits and incubation times, while, for all other performance metrics, the highest values were observed after 6 h of incubation. Across microorganisms and incubation times, the calculated 95% CI for Se showed wide ranges, all extending up to 1.00, whereas the 95% CI for Sp were narrower and varied slightly between 6 and 9 h. The value found for Cohen’s kappa coefficient (κ) was also calculated to assess the level of agreement between the LAMP method and the culture-based gold standard after both 6 and 9 h of incubation with the pre-enrichment medium (Table 3).

## 4. Discussion

In recent years, laboratory-based microbiology has undergone a significant transformation, driven by the increasing adoption of rapid diagnostic methods [35]. In clinical settings, molecular techniques such as polymerase chain reaction (PCR), real-time PCR (qPCR), multiplex PCR, metagenomic, next-generation sequencing (NGS), and isothermal amplification methods have revolutionized diagnostic workflows [36,37,38]. These technologies enable the rapid detection and identification of pathogens directly from clinical specimens, often within hours, compared to the several days typically required by conventional culture-based approaches [39]. Beyond clinical diagnostics, rapid microbiological methods are also gaining relevance in environmental monitoring, particularly in food safety and water monitoring, where timely detection of microbial contamination is essential for infection control and quality assurance [40,41]. In hospital environments, the ability to detect contamination quickly and accurately is critical for preventing HAIs [42]. This need is further amplified by the global rise in antimicrobial resistance (AMR), which has been recognized as one of the most urgent public health threats [43]. The WHO and other international bodies have emphasized the importance of rapid diagnostics in combating AMR, as they allow for early identification of resistant strains and reduce the misuse of broad-spectrum antibiotics [44,45]. Within this context, prevention strategies become increasingly important, and environmental surveillance plays a key role [46]. The implementation of LAMP as a monitoring tool for hospital surfaces could represent a valuable strategy for early screening, enabling timely interventions and reducing the risk of pathogen transmission.

A previous study by Marino et al. [29] demonstrated that the three LAMP kits used in the present work, originally developed for water testing, can be successfully adapted for microbiological surface monitoring. Conducted under controlled laboratory conditions, that study primarily aimed to identify the minimum incubation time required to obtain reliable and reproducible results, comparable to those achieved with the gold-standard culture method. Starting from the 18-h incubation period recommended by the manufacturer, the research identified 6 and 9 h of sample incubation in the pre-enrichment medium as optimal for kit performance [29]. Building on these findings, the present study evaluated both incubation times in real hospital environments, where variables such as surface material, cleaning protocols, microbial stress, and human impact may significantly influence detection outcomes.

Compared to the previous study, some discrepancies were observed. In the present work, Se remained consistently at 1.00 for all pathogens and incubation times, while Sp values were slightly lower, ranging from 0.89 for *Enterococcus* spp. and 0.93 for *P. aeruginosa*. These data are in contrast with the earlier laboratory-based study, where Se varied depending on the incubation time, and Sp remained stable. The difference may be attributed to the low number of positive samples detected by the reference culture-based method, which likely underestimates the true presence of pathogens. Indeed, in real-world conditions, microbial growth on culture media can be inhibited by several factors, including desiccation, presence of disinfectant residues, competition, and sublethal stress, making culture-based detection less reliable [47,48].

The 95% CI values calculated for Se and Sp were consistent with this observation. Although sensitivity was high across all evaluated conditions, the corresponding 95% CI were relatively wide (e.g., from 0.54–1.00 for *P. aeruginosa* to 0.81–1.00 for *Enterococcus* spp.), indicating substantial statistical uncertainty due to the limited number of positive samples. Thus, despite the absence of false negatives, the true Se of LAMP may be lower than the point estimate suggested. In contrast, the 95% CI for Sp were considerably narrower and consistently fell within the range 0.82–0.96 for all microorganisms and both incubation times, reflecting a more precise and stable estimate, consistent with the larger number of negative samples recovered. Overall, these results indicate that larger datasets, particularly those including a higher number of positive cases, would allow for more precise and reliable estimates of sensitivity.

In general, molecular methods such as LAMP can indeed detect DNA from VBNC organisms, offering a more sensitive and comprehensive assessment of contamination [49]. This is supported by studies showing that LAMP assays can identify pathogens even when traditional culture fails, particularly in food and clinical samples [50,51]. Furthermore, other studies have reported high Se but slightly lower Sp for LAMP compared to culture-based methods, depending on the pathogen and sample type [52,53]. However, this result may also be influenced by a typical limitation of molecular techniques, which can detect DNA even from non-viable or dead cells [54]. Although this could lead to obtaining false positive results, especially in environmental monitoring, it is important to consider the practical implications. Even if the specificity of LAMP is slightly lower, the risk of missing a true contamination (false negative) is reduced. In this context, a precautionary approach is preferable: performing an additional sanitization in response to a potentially false positive is a safer choice than overlooking a contamination that could pose a real risk.

Regarding the distribution of positive results among the different hospital risk areas, interestingly, the culture-based method identified pathogens only in MR and LR areas, while LAMP detected contamination also in HR zones. This finding is consistent with previous studies suggesting that MR and LR environments may harbor significant microbial contamination, despite being considered less critical. In fact, non-critical areas such as general wards, outpatient clinics, and waiting rooms often involve high patient turnover and frequent surface contact, which can contribute to environmental contamination detectable by culture-based methods [55,56]. Conversely, HR areas are subject to stricter disinfection protocols, which may reduce the recovery of viable organisms by culture. However, molecular methods like LAMP can still detect microbial DNA, including non-viable cells, allowing for broader contamination detection even in these highly sanitized settings. Similarly, the VHR areas showed no contamination with any method, likely due to stricter cleaning and disinfection protocols [57].

In general, for all environments, the proportion of positive samples detected by LAMP was higher than that detected by the culture method, in line with other comparative studies between molecular and traditional techniques [58,59].

Regarding Cohen’s kappa, *P. aeruginosa* showed the lowest agreement value among the three targets at both incubation times (κ = 0.52; κ = 0.42). This result can be explained by several methodological and biological factors. Culture detected very few positive samples (*n* = 6), whereas LAMP identified a substantially higher number of positives (*n* = 16; *n* = 20), increasing the proportion of LAMP-positive/culture-negative results, and mathematically reducing the κ coefficient despite a sensitivity of 1.00. This pattern is consistent with the well-documented ability of *P. aeruginosa* to enter a VBNC state on dry or disinfected surfaces, leading to underestimation by culture [60]. Finally, the low prevalence of culture-positive samples is known to reduce the stability and interpretability of κ statistics [61]. Taken together, these factors explain the moderate κ value without indicating poor analytical performance of the LAMP method.

Considering the different incubation times tested, for all tested kits, the overall performance metrics and Cohen’s kappa values indicated that optimal diagnostic performance was already achieved after 6 h of incubation, suggesting that this shorter time is sufficient for reliable screening in real-world conditions. This is particularly relevant for hospital workflows, where rapid turnaround times can support timely decision-making and targeted cleaning interventions, and help to best plan the disinfection treatment scheduling.

Beyond its analytical performance, the LAMP approach offers practical and economic advantages that enhance its suitability for routine environmental surveillance. In our study, the complete LAMP workflow required only 6–9 h, whereas culture and subsequent identification required several days, resulting in a substantially shorter turnaround time. When compared with the culture-based method, LAMP generally involves higher per-test reagent costs; however, culture requires prolonged incubation, multiple processing steps, and access to specialized, high-cost identification systems (e.g., MALDI-TOF), which further increase labor demands, consumable consumption, and overall operational costs. The reduced hands-on time and simplified workflow of LAMP therefore have the potential to offset its analytical cost, particularly in settings where rapid decision-making can prevent extended contamination or additional cleaning cycles.

Additionally, compared with qPCR, which remains the most widely used molecular method for bacterial detection, LAMP also offers notable financial and logistical advantages. qPCR requires complex thermocyclers, controlled laboratory environments, and trained personnel, contributing to higher equipment and maintenance costs. In contrast, LAMP operates under isothermal conditions and can be performed using compact, portable devices, substantially lowering the infrastructural and technical requirements for implementation. These considerations suggest that LAMP may represent a more cost-effective and easily deployable option for on-site monitoring, enabling faster responses and more frequent assessments in healthcare settings.

Although LAMP cannot totally replace the culture-based method in hospital environmental surveillance, which remains essential for pathogen isolation, antibiotic susceptibility testing, and outbreak or cluster epidemiological investigation, it could be integrated as a rapid screening tool. Thanks to its speed and high sensitivity, LAMP is well-suited to support routine monitoring, optimize disinfection protocols, validate cleaning procedures, and help schedule hospital activities more efficiently. Its ability to provide fast results makes it ideal for early detection of contamination, guiding confirmatory testing, and preventive actions. Moreover, the rapid turnaround time of LAMP could enable more frequent monitoring of hospital environments. This could reduce the reliance on more aggressive disinfection protocols that involve chemical agents with significant environmental impact, potentially contributing to antimicrobial resistance and influencing ecological differentiation [62]. A more frequent and targeted monitoring strategy could allow for a more balanced and sustainable approach to disinfection.

## 5. Conclusions

LAMP represents a promising alternative to culture-based methods for microbiological monitoring of hospital surfaces. Its rapid detection capabilities and high sensitivity can enhance and support infection control strategies, reducing the incidence of HAIs.

## Figures and Tables

**Table 1:** Number of positive samples for the different detection methods on a total of 145 hospital surface samples.

	Positive Samples/Total (%)		
	Cultural Method	LAMP	
		6 h	9 h
*P. aeruginosa*	6/145 (4%)	16/145 (11%)	20/145 (14%)
*S. aureus*	10/145 (7%)	21/145 (14%)	21/145 (14%)
*Enterococcus* spp.	18/145 (12%)	30/145 (21%)	32/145 (22%)

For *P. aeruginosa* and *Enterococcus* spp., the highest detection rates were obtained using LAMP after 9 h of incubation. In contrast, *S. aureus* showed the highest detection rate with LAMP regardless of incubation time.

**Table 2:** Performance metrics of LAMP detection kits after different incubation times (6 and 9 h). Sensitivity (Se), specificity (Sp), with 95% CI, accuracy (Acc), positive predictive value (PPV), negative predictive value (NPV), F1-score (F1), and balanced accuracy (Bal Acc).

LAMP Detection Kit	Time (h)	Se	95% CI	Sp	95% CI	Acc	PPV	NPV	F1	Bal Acc
***P. aeruginosa***	**6**	1.00	(0.54–1.00)	0.93	(0.87–0.96)	0.93	0.38	1.00	0.55	0.96
	**9**	1.00	(0.54–1.00)	0.90	(0.83–0.94)	0.90	0.30	1.00	0.46	0.95
***S. aureus***	**6**	1.00	(0.69–1.00)	0.92	(0.85–0.95)	0.92	0.48	1.00	0.65	0.96
	**9**	1.00	(0.69–1.00)	0.92	(0.85–0.95)	0.92	0.48	1.00	0.65	0.96
***Enterococcus*** **spp.**	**6**	1.00	(0.81–1.00)	0.91	(0.84–0.95)	0.92	0.60	1.00	0.75	0.95
	**9**	1.00	(0.81–1.00)	0.89	(0.82–0.94)	0.90	0.56	1.00	0.72	0.94

**Table 3:** Cohen’s kappa coefficient (κ) values for each LAMP detection kit after 6 and 9 h of incubation.

	Cohen’s Kappa Coefficient (κ)	
LAMP Detection Kit	6 h	9 h
*P. aeruginosa*	0.52	0.42
*S. aureus*	0.61	0.61
*Enterococcus* spp.	0.70	0.67

For *P. aeruginosa* and *Enterococcus* spp., the highest κ values were observed after 6 h of incubation, with the overall highest agreement for *Enterococcus* spp. (κ = 0.70). In contrast, *S. aureus* showed identical κ values at both time points.

## Data Availability

All relevant data are provided in the manuscript. The corresponding author can provide additional data as a reasonable request.
